# Natural History of Quantitative Fatty Infiltration and 3D Muscle Volume After Nonoperative Treatment of Symptomatic Rotator Cuff Tears: A Prospective MRI Study of 79 Patients

**DOI:** 10.2106/JBJS.23.01083

**Published:** 2024-02-22

**Authors:** Bettina Hochreiter, Christoph Germann, Georg C. Feuerriegel, Reto Sutter, Farah Selman, Maximilian Gressl, Eugene T. Ek, Karl Wieser

**Affiliations:** 1 Department of Orthopaedics, Balgrist University Hospital, University of Zurich, Zurich, Switzerland; 2 Department of Radiology, Balgrist University Hospital, University of Zurich, Zurich, Switzerland; 3 Melbourne Orthopaedic Group, Melbourne, Victoria, Australia; 4 Department of Surgery, Monash Medical Center, Monash University, Melbourne, Victoria, Australia

## Abstract

**Background:**

The severity of fatty infiltration (FI) predicts the treatment outcome of rotator cuff tears. The purpose of this investigation was to quantitatively analyze supraspinatus (SSP) muscle FI and volume at the initial presentation and after a 3-month minimum of conservative management. We hypothesized that progression of FI could be predicted with initial tear size, FI, and muscle volume.

**Methods:**

Seventy-nine shoulders with rotator cuff tears were prospectively enrolled, and 2 magnetic resonance imaging (MRI) scans with 6-point Dixon sequences were acquired. The fat fraction within the SSP muscle was measured on 3 sagittal slices, and the arithmetic mean was calculated (FI^SSP^). Advanced FI^SSP^ was defined as ≥8%, pathological FI^SSP^ was defined as ≥13.5%, and relevant progression was defined as a ≥4.5% increase in FI^SSP^. Furthermore, muscle volume, tear location, size, and Goutallier grade were evaluated.

**Results:**

Fifty-seven shoulders (72.2%) had normal FI^SSP^, 13 (16.5%) had advanced FI^SSP^, and 9 (11.4%) had pathological FI^SSP^ at the initial MRI scan. Eleven shoulders (13.9%) showed a ≥4.5% increase in FI^SSP^ at 19.5 ± 14.7 months, and 17 shoulders (21.5%) showed a ≥5-mm^3^ loss of volume at 17.8 ± 15.3 months. Five tears (7.1%) with initially normal or advanced FI^SSP^ turned pathological. These tears, compared with tears that were not pathological, had significantly higher initial mediolateral tear size (24.8 compared with 14.3 mm; p = 0.05), less volume (23.5 compared with 34.2 mm^3^; p = 0.024), more FI^SSP^ (9.6% compared with 5.6%; p = 0.026), and increased progression of FI^SSP^ (8.6% compared with 0.5%; p < 0.001). An initial mediolateral tear size of ≥20 mm yielded a relevant FI^SSP^ progression rate of 81.8% (odds ratio [OR], 19.0; p < 0.001). Progression rates of 72.7% were found for both initial FI^SSP^ of ≥9.9% (OR, 17.5; p < 0.001) and an initial anteroposterior tear size of ≥17 mm (OR, 8.0; p = 0.003). Combining these parameters in a logistic regression analysis led to an area under the receiver operating characteristic curve (AUC) of 0.913. The correlation between FI^SSP^ progression and the time between MRI scans was weak positive (ρ = 0.31).

**Conclusions:**

Three risk factors for relevant FI progression, quantifiable on the initial MRI, were identified: ≥20-mm mediolateral tear size, ≥9.9% FI^SSP^, and ≥17-mm anteroposterior tear size. These thresholds were associated with a higher risk of tear progression: 19 times higher for ≥20-mm mediolateral tear size, 17.5 times higher for ≥9.9% FI^SSP^, and 8 times higher for ≥17-mm anteroposterior tear size. The presence of all 3 yielded a 91% chance of ≥4.5% progression of FI^SSP^ within a mean of 19.5 months.

**Level of Evidence:**

Diagnostic Level II. See Instructions for Authors for a complete description of levels of evidence.

Nonoperatively treated rotator cuff tears run the risk of developing progressive fatty muscle infiltration (FI) over time. Successful rotator cuff repair has been shown to halt, but not reverse, FI^1-5^. It is known that tears do enlarge over time^6-11^ and that enlargement is associated with the progression of FI in both a clinical setting^1,6,7,10,12^ and an experimental setting^13^. The severity of FI predicts the outcome of operative^14-16^ and nonoperative treatment^17^.

To date, the indications for operative rotator cuff tear management still vary widely, and precise knowledge about the natural history of rotator cuff tears is therefore crucial when counseling patients with regard to the optimal timing for surgical intervention, especially if a trial of nonoperative management is considered. Although there are studies in which authors have reported on rotator cuff tear progression with respect to size or FI assessed by sonography or qualitative and/or semiquantitative magnetic resonance imaging (MRI) scans^1,6-10,18-23^, none has looked at the natural progression of FI with quantitative MRI measurements.

Despite the ease of use of the Goutallier classification^24^, adapted by Fuchs et al.^25^, which is based on a single computed tomographic (CT) or MRI slice, it is not without substantial limitations. It is a subjective, single-plane, qualitative grading system with only moderate interobserver and intraobserver reliability^26-28^. Recently, technological advancements have allowed for quantitative analysis of the muscle fat fraction (FF) with Dixon sequences^2,29-31^ and 3-dimensional (3D) volumetric analysis^2,31,32^ on MRI scans. Single-slice measurements in 2-dimensional (2D) Y-views are appealing, especially in the clinical setting, but, as intramuscular fat distribution is inhomogeneous^33-35^, the measurements hardly correlate with the global 3D FF^28,33,36-38^. For this reason, Xu et al. proposed a modified assessment tool^39^ consisting of the mean of 3 FF measurements on 6-point Dixon sagittal images: the scapular Y-view and 2 adjacent slices. They showed that this mean FF correlates strongly with the 3D volumetric global FF of the entire supraspinatus (SSP) muscle^39^.

The purpose of this investigation was to quantitatively and longitudinally analyze the mean SSP muscle FI on MRI Dixon sequences and 3D volume at the initial presentation and after a 3-month minimum of nonoperative management. We hypothesized that the progression of FI could be predicted with the initial tear size, FI, and muscle volume.

## Materials and Methods

### Patients

Ethical review board approval was obtained prior to the initiation of the study (KEK-Zürich, Basec No. 2018-02285). Between July 2016 and February 2023, patients who met the following inclusion criteria were prospectively enrolled: a symptomatic full-thickness rotator cuff tear confirmed with magnetic resonance (MR) arthrography and determined by an orthopaedic surgeon and musculoskeletal radiologist at the initial visit, at least 2 MRI scans with 6-point Dixon sequences and high-quality sagittal images, at least 3 months of follow-up between the 2 MRI scans, and nonoperative treatment until the second MRI. Nonoperative treatment included analgesia, subacromial or glenohumeral cortisone injections, and/or physiotherapy. Exclusion criteria were previous shoulder surgery, glenohumeral osteoarthritis or inflammatory arthritis, and partial-thickness tears.

Conservative management was the preferred initial treatment option for chronic or atraumatic rotator cuff tears, especially in older and less active patients. Rotator cuff repair was preferred for traumatic full-thickness tears in younger (≤50 years) and active patients. However, some patients declined a surgical procedure for personal reasons or comorbidities and were treated nonoperatively. Patients were asked to return to the clinic for a follow-up MRI scan after 3, 6, or ≥12 months, depending on the surgeons’ assessment of the risk of tear progression. Larger tears with involvement of the anterior cable in younger patients tended to have follow-up within 12 months. Patients had follow-up regardless of whether they were or were not experiencing pain or symptoms, but were also told to return earlier if they noticed an increase in pain^6,7,9^ or a decrease in strength^22^, both potentially indicating faster tear progression.

**Fig. 1:**
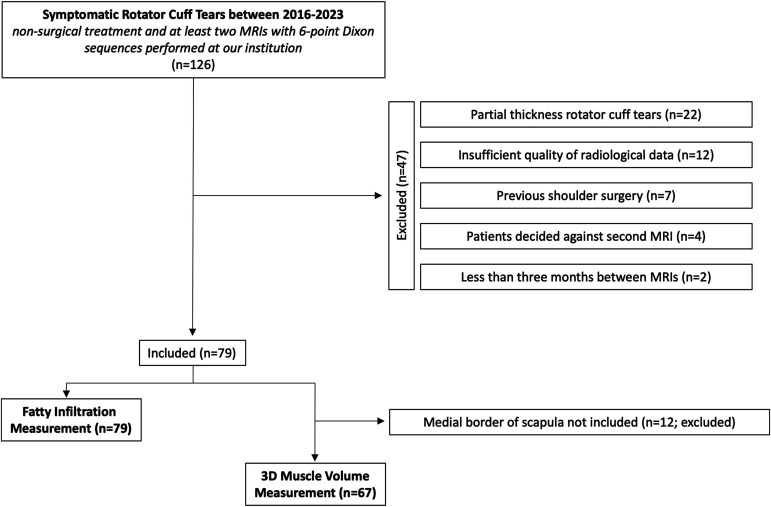
STROBE (Strengthening the Reporting of Observational Studies in Epidemiology) flowchart.

Seventy-nine rotator cuff tears (in 36 men and 41 women [2 patients had bilateral tears], with a mean age of 61 years [range, 36 to 81 years]) were analyzed (Fig. 1). The mean follow-up was 14.5 months (range, 3 to 55 months).

### MRI

All patients underwent an MRI scan in a 1.5-T (MAGNETOM Avanto Fit; Siemens Healthineers) or a 3-T scanner (MAGNETOM Skyra Fit; Siemens Healthineers) with a dedicated 16-channel phased-array shoulder coil (see Appendix Table 1 for MRI parameters). The MRI protocol was identical for all patients and both imaging time points. For quantification of the SSP muscle FF, a sagittal oblique 6-point Dixon sequence was acquired, from which in-phase, out-of-phase, water-only, and fat-only images were generated. The slice thickness was 3 mm. Two fellowship-trained musculoskeletal radiologists (C.G. and G.C.F.) evaluated all MRI scans independently and were blinded to clinical information.

### Mean FI Measurement of SSP Muscle

**Fig. 2:**
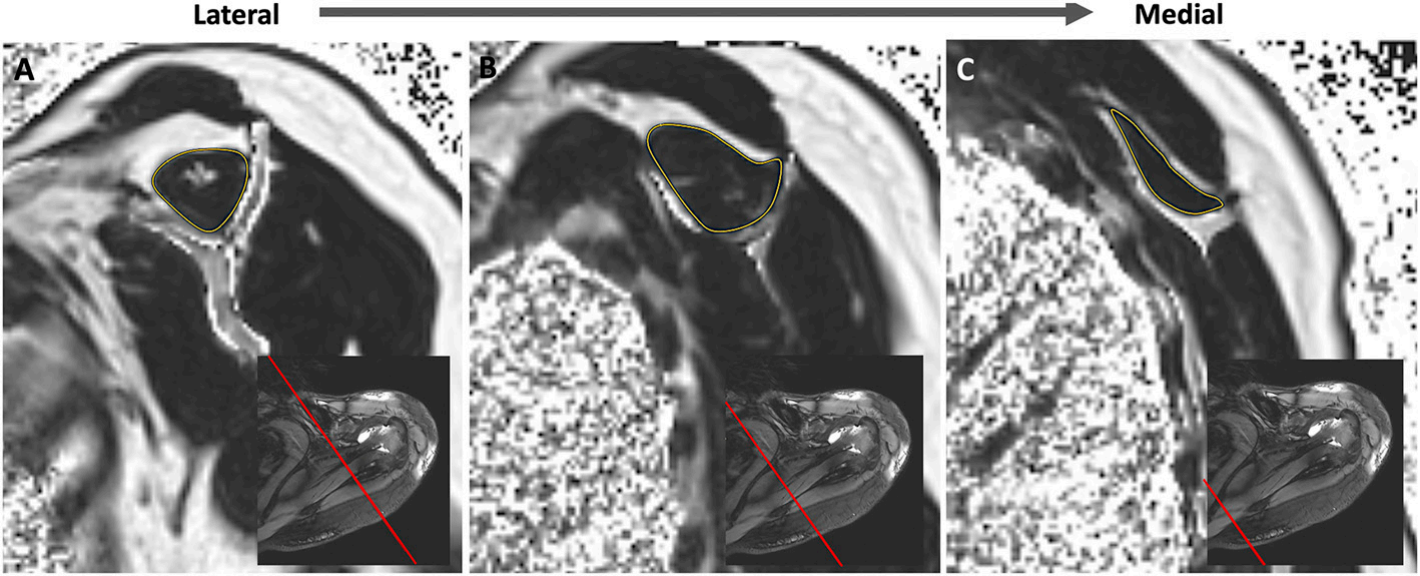
The region of interest (yellow) was placed at the inner margin of the SSP muscle on the most lateral slice showing the scapular Y (**Fig. 2-A**), halfway between the Y-view and the medial origin of the muscle (**Fig. 2-B**), and on the most medial image showing the SSP origin (**Fig. 2-C**). The red line on each inset shows the location of the selected slice on the axial plane. The arithmetic mean fat fraction (FISSP) was determined by averaging the fat fraction of these 3 slices.

The quantitative evaluation of FF was performed by manually placing a region of interest at the inner margin of the SSP muscle, avoiding inclusion of the adjacent perimuscular fat or bone, on 3 slices: the most lateral slice showing the scapular Y, halfway between the Y-view and the medial origin of the SSP, and the most medial image showing the SSP origin (Fig. 2). The Dixon sequence allowed separation of the signal for each voxel into the signal intensity of fat and that of water, from which the fat percentage could be calculated for that voxel. The following equation was used to calculate the intramuscular FF^31,40^:

$$Fat\;\%=\frac{Fat\;signal}{Fat\;signal+water\;signal}\times 100\%$$

The arithmetic mean FF (FI^SSP^) was determined by averaging the 3 measurements. Normal FI^SSP^ was defined as <8% FF, based on a previous study that showed that the normal mean FI^SSP^ in healthy participants, measured with 6-point Dixon MRI scans, was 7.7% and the upper limit of the 95% confidence interval (CI) was 8%^31^. Therefore, advanced FI^SSP^ was defined as 8% to <13.5% FF. Pathological FI^SSP^ was defined as ≥13.5% FF, based on previous studies showing that Goutallier grades of ≥2 were associated with a definitive loss of muscle function^4,41^: in our study, grade 2 corresponded to a mean FI^SSP^ of 14.8%, and the lower limit of the 95% CI was 13.5% (Table I). Table I shows the number and percentages of SSPs classified as normal, advanced, or pathological, as well as the corresponding mean FI^SSP^ at each MRI scan. The relevant progression of FI^SSP^ was defined as a ≥4.5% increase in mean FI^SSP^. An increase of ≥4.5% FI, in an SSP initially classified as normal (mean FI^SSP^ of 4.8% in our study), would lead to it being classified as advanced according to our classification system, as the lower limit of the 95% CI of FI^SSP^ in the advanced group was 9.3%. In other words, 4.5% is the difference between the mean value of FI^SSP^ in the normal group and the lower limit of the 95% CI in the advanced group.

**TABLE I:** SSPs Grades in Initial and Follow-up MRI Scans and the Corresponding FI^SSP^ Values

Grade*	Initial MRI†	FI^SSP^‡	Follow-up MRI†	FI^SSP^‡
0, normal	57 (72.2%)	4.8% ± 1.35%	47 (59.5%)	4.9 ± 1.6
1, advanced	13 (16.5%)	10.8% ± 1.9%	18 (22.8%)	9.6 ± 1.1
2, pathological	9 (11.4%)	18.8% ± 5.4%	14 (17.7%)	22.1 ± 6.2

* The FI^SSP^ (fatty infiltration of the supraspinatus) values defining the 3 grades are: <8%, normal; 8% to <13.5%, advanced; and ≥13.5%, pathological.

† The values are given as the number of shoulders, with the percentage in parentheses.

‡ The values are given as the mean and the standard deviation.

### 3D SSP Muscle Volume Measurement

SSP muscle volume was only measured on scans that included the medial border of the scapula on coronal and/or sagittal slices (n = 67) (see Appendix for a detailed description). The progression of atrophy was arbitrarily defined as a decrease in volume of ≥5 mm^3^.

### Other MRI Parameters

Tear location, size calculated as described by DeOrio and Cofield^42^, and rotator cable involvement were evaluated. The mediolateral extent of the tear on coronal images (tendon retraction) and anteroposterior extent of the tear on sagittal images were measured (in millimeters) and the maximum in each direction was defined as the tear size. The tear area was calculated (in mm^2^) by multiplying mediolateral and anteroposterior tear sizes. An increase of ≥5 mm in either mediolateral or anteroposterior extension was defined as an increase in tear size^10,23^. Tendon length was measured. FI was evaluated qualitatively according to the method of Fuchs et al.^25^, which was based on the study by Goutallier et al.^24^.

### Statistical Analysis

We created 2 groups according to the MRI time point and the progression of FI^SSP^. Continuous outcomes were compared with the unpaired Student t test, Welch t test, or Mann-Whitney-U test; discrete outcomes were compared with the chi-square test or Fisher exact test. A p value of ≤0.05 was considered significant. The correlation between the progression of FI^SSP^ and the time between MRI scans was assessed with the Spearman coefficient.

A binary logistic regression analysis was conducted to determine the most significant associations with relevant progression of FI^SSP^. Odds ratios (ORs) are presented with 95% CIs. The goodness of fit of the logistic regression models was assessed via the Hosmer-Lemeshow test. Receiver operating characteristic (ROC) curves were created to determine cutoff values for the parameters that most significantly influenced the model. The area under the curve (AUC), 95% CIs, and optimal threshold values that maximized the Youden index were calculated. The AUC was categorized as acceptable (0.7 to <0.8), excellent (0.8 to <0.9), or outstanding (0.9 to 1)^43^.

Intraclass correlation coefficient (ICC) estimates and their 95% CIs were calculated to assess agreement between the 2 observers. Statistical analysis was performed with EasyMedStat and GraphPad Prism (GraphPad Software) (see the Appendix for a detailed description).

## Results

**Fig. 3:**
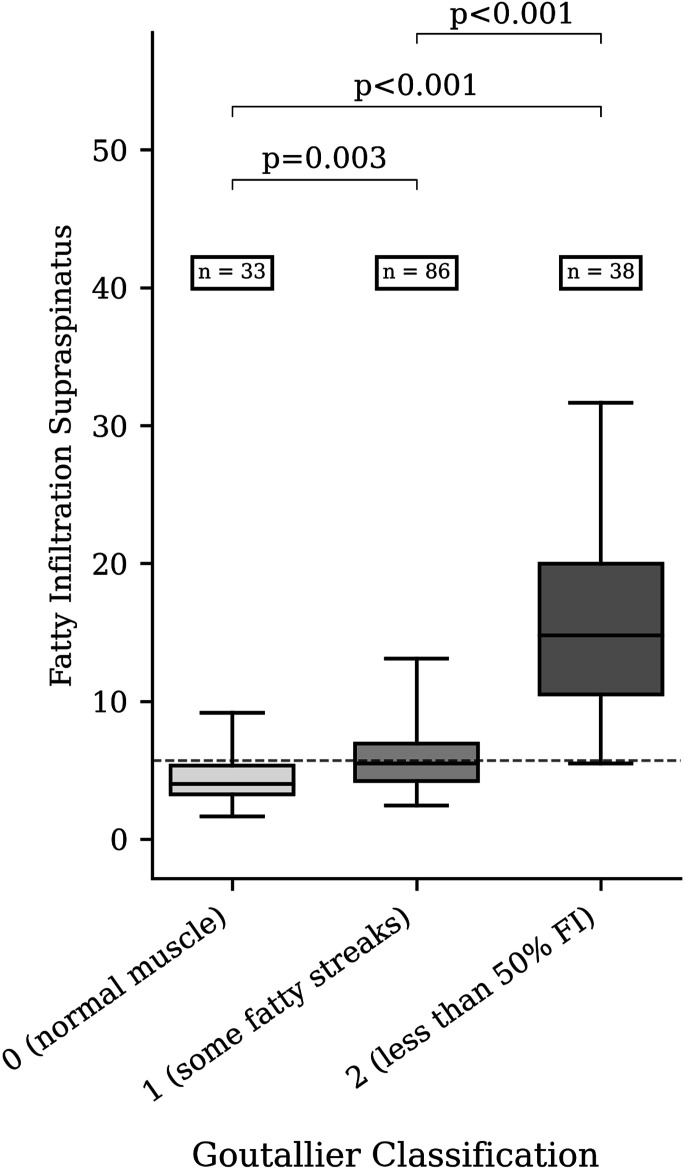
FISSP according to the Goutallier classification. The box is the interquartile range, and the line within the box is the median. The whiskers indicate the range.

The measured MRI parameters are shown in Table II. The median FI^SSP^ was 4% (range, 1.7% to 9.2%) for SSPs with Goutallier grade 0, 5.5% (range, 2.4% to 13%) for Goutallier grade 1, and 14.8% (range, 5.5% to 31.7%) for Goutallier grade 2 (Fig. 3). One SSP was graded as Goutallier grade 3; the corresponding FI^SSP^ was 36.7%. A weak positive correlation was found between the progression of FI^SSP^ and the time between MRI scans (ρ = 0.31; p = 0.005).

**TABLE II:** Comparison of MRI Parameters Between Initial and Follow-up MRI Scans*

Parameter	Initial MRI† (N = 79)	Follow-up MRI† (N = 79)	Difference‡	P Value
FI^SSP^ *(%)*	7.4 ± 5.2 (1.7 to 31.6)	8.9 ± 7 (1.8 to 36.7)	1.6 ± 3.4 (0.8 to 2.3)	0.208
Muscle volume *(mm*^*3*^*)* (n = 67)	32.5 ± 10 (16.2 to 60.6)	29.5 ± 9.5 (8.6 to 53.6)	−3 ± 4.6 (−4.1 to −1.8)	0.083
Tear location				0.68
Anterior third	14 (17.7%)	8 (10.1%)		
Anterior and middle thirds	35 (44.3%)	41 (51.9%)		
Middle third	4 (5.1%)	3 (3.8%)		
Middle and posterior thirds	23 (29.1%)	24 (30.4%)		
Posterior third	3 (3.8%)	3 (3.8%)		
Anterior tear involvement				>0.999
Yes	48 (60.8%)	49 (62.0%)		
No	31 (39.2%)	30 (38.0%)		
Cofield classification				0.449
Large, 3 to 5 cm	11 (13.9%)	16 (20.3%)		
Medium, 1 to <3 cm	54 (68.4%)	53 (67.1%)		
Small, <1 cm	14 (17.7%)	10 (12.7%)		
Tendon length *(mm)*	24.7 ± 7.2 (7.0 to 38.0)	23.01 ± 7.3 (7.0 to 41.0)	−1.7 ± 4.4 (−2.7 to −0.7)	0.102
Anteroposterior tear size *(mm)*	15 ± 7.5 (5.0 to 42.0)	16.5 ± 8.1 (5.0 to 43.0)	1.5 ± 4.8 (0.5 to 2.6)	0.216
Mediolateral tear size *(mm)*	16.3 ± 9.6 (5.0 to 46.0)	18.5 ± 10.6 (6.0 to 50.0)	2.2 ± 5.2 (1 to 3.3)	0.201
Tear area *(mm*^*2*^*)*	289.2 ± 293.5 (25.0 to 1,302.0)	356.8 ± 341.2 (40.0 to 1,400.0)	67.6 ± 171.3 (29.2 to 105.9)	0.15
Goutallier classification				0.068
0, normal muscle	22 (27.9%)	11 (13.9%)		
1, some fatty streaks	42 (53.2%)	44 (55.7%)		
2, <50% FI	15 (18%)	23 (29.1%)		
3, ≥50% FI	0 (0.0%)	1 (1.3%)		

* FI = fatty infiltration, and SSP = supraspinatus.

† The values are given as the mean and the standard deviation, with the range in parentheses, or as the number of shoulders, with the percentage in parentheses.

‡ The values are given as the mean and standard deviation, with the 95% CI in parentheses.

### Progression of FI, Atrophy, and Tear Size

Fifty-seven shoulders (72.2%) initially had normal FI^SSP^. Of those, 47 (82.5%) remained normal, 8 (14%) progressed to advanced, and 2 (3.5%) progressed to pathological. Thirteen shoulders (16.5%) initially had advanced FI^SSP^. Of those, 10 (76.9%) remained advanced and 3 (23.1%) progressed to pathological. Nine shoulders (11.4%) had pathological FI^SSP^ at both time points (Tables I and III). Thus, 5 tears (7.1%) with initially normal or advanced FI^SSP^ became pathological. These tears had significantly higher mediolateral tear size, less muscle volume, more FI^SSP^, and increased progression of FI^SSP^ (Table IV).

**TABLE III:** SSPs That Remained the Same or Progressed According to Our Classification*

Change in Classification	SSPs†	Difference in Time‡ *(mo)*
Remained normal	47 (82.5%) of 57	
Remained advanced	10 (76.9%) of 13	
Remained pathological	9 (100%) of 9	
Normal to advanced	8 (14.0%) of 57	19.7 ± 17
Advanced to pathological	3 (23.1%) of 13	11.3 ± 8
Normal to pathological	2 (3.5%) of 57	41.8

* Normal: <8% FI^SSP^; advanced: 8% to <13.5% FI^SSP^; and pathological: ≥13.5% FI^SSP^. FI = fatty infiltration, and SSP = supraspinatus.

† The values are given as the number of shoulders, with the percentage in parentheses.

‡ The values are given as the mean with or without the standard deviation.

**TABLE IV:** Comparison of Tears That Did Not Have Pathological FI^SSP^ (≥13.5%) at Either Time Point, That Became Pathological at the Follow-up MRI, and That Already Had Pathological FI^SSP^ at the Initial MRI Scan*

Variable	FI^SSP^ Status of Tear†			P Value‡	
	Remained Not Pathological at Follow-up (N = 65)	Became Pathological at Follow-up (N = 5)	Remained Pathological (N = 9)	Remained Not Pathological Vs. Became Pathological	Remained Not Pathological Vs. Initially Pathological
Age *(yr)*	59.9 ± 9.4 (36.0 to 81.0)	65.0 ± 4.5 (60.0 to 72.0)	63.8 ± 8.4 (52.0 to 76.0)	NS	NS
Initial anteroposterior tear size *(mm)*	13.5 ± 5.5 (5.0 to 27.0)	21.4 ± 12.6 (11.0 to 42.0)	22.4 ± 11 (10.0 to 40.0)	NS	NS
Initial mediolateral tear size *(mm)*	14.3 ± 7.9 (5.0 to 46.0)	24.8 ± 12.3 (11.0 to 36.0)	26.0 ±11.8 (8.0 to 46.0)	**0.05**	**0.003**
Initial muscle volume§ *(mm*^*3*^*)*	34.2 ± 10 (19.9 to 60.6)	23.5 ± 4.8 (18.4 to 28.3)	26.1 ± 6.6 (16.2 to 35.2)	**0.024**	**0.03**
Initial FI^SSP^ *(%)*	5.6 ± 2.5 (1.7 to 13.4)	9.6 ± 3.5 (5.4 to 13.1)	18.8 ± 5.4 (14.7 to 31.6)	**0.026**	**<0.001**
Follow-up FI^SSP^ *(%)*	6.1 ± 7 (1.8 to 11.7)	18.1 ± 4.4 (14.7 to 25.3)	24.3 ± 6.1 (17.7 to 36.7)	**0.035**	**0.002**
Progression of FI^SSP^ *(%)*	0.5 ± 1.7 (−3.7 to 5.5)	8.6 ± 6 (4.0 to 18.7)	5.5 ± 3.8 (1.0 to 11.7)	**<0.001**	**<0.001**
Loss of muscle volume§ *(mm*^*3*^*)*	−3.3 ± 4.2 (−15.4 to 5.7)	0.7 ±7.4 (−9.8 to 7.5)	−2.4 ± 5.4 (−15.3 to 3.3)	NS	NS
Time between MRI scans *(min)*	13.7 ± 11.8 (3 to 49.3)	23.5 ± 20 (5 to 55.1)	15.4 ± 7.8 (6.2 to 28.4)	NS	NS

* FI = fatty infiltration, SSP = supraspinatus, and NS = not significant.

† The values are given as the mean and the standard deviation, with the range in parentheses.

‡ Significant values are shown in bold.

§ There were 54 shoulders with data in the group that remained not pathological, 4 shoulders in the group that became pathological, and 9 shoulders in the initially pathological group.

Eleven shoulders (13.9%) showed relevant FI^SSP^ progression at a mean (and standard deviation) of 19.5 ± 14.7 months (Table V). Seventeen shoulders (21.5%) showed ≥5-mm^3^ loss of SSP muscle volume at a mean of 17.8 ± 15.3 months; the mean change in SSP muscle volume was −9.2 ± 3.3 mm^3^ compared with −0.8 ± 2.6 mm^3^ in the remaining shoulders (p < 0.001). Another 17 shoulders (21.5%) showed ≥5-mm tear size enlargement at a mean of 22.4 ± 16.2 months; the mean change in anteroposterior tear size was 7.1 ± 5.2 mm compared with 0.0 ± 3.3 mm in the remaining shoulders (p < 0.001), and the mean change in mediolateral tear size was 8.2 ± 7.7 mm compared with 0.5 ± 2.3 mm in the remaining shoulders (p < 0.001).

**TABLE V:** Comparison of Groups with and without FI^SSP^ Progression of ≥4.5%*

	Initial MRI Scan			Follow-up MRI Scan			Difference		
	FI^SSP^ Progression ≥4.5%†		P Value‡	FI^SSP^ Progression ≥4.5%†		P Value‡	FI^SSP^ Progression ≥4.5%†		P Value‡
	No (N = 68)	Yes (N = 11)		No (N = 68)	Yes (N = 11)		No (N = 68)	Yes (N = 11)	
Age *(yr)*	59.9 ± 9.4 (36.0 to 81.0)	65.2 ± 5.9 (53.0 to 74.0)	0.073						
Follow-up *(mo)*							13.7 ±11.6 (3 to 49.3)	19.5 ± 14.7 (4.2 to 55.1)	0.118
Cofield classification			**<0.001**						
Large, 3 to 5 cm	5 (7.4%)	6 (54.5%)							
Medium, 1 to <3 cm	49 (72.1%)	5 (45.5%)							
Small, <1 cm	14 (20.6%)	0 (0.0%)							
FI^SSP^ *(%)*	6.3 ± 3.8 (1.7 to 21.3)	13.8 ± 7.7 (4.1 to 31.6)	**<0.001**	6.8 ± 4.1 (1.8 to 22.3)	21.9 ± 7.8 (9.6 to 36.7)	**<0.001**	0.5 ± 1.6	8.1 ± 4.3	**<0.001**
Muscle volume§ *(mm*^*3*^*)*	33.7 ± 10 (19.8 to 60.6)	24.8 ± 6.5 (16.2 to 35.2)	**0.013**	30.58 ± 9.3 (17.9 to 53.6)	22.6 ± 8.1 (8.6 to 34.2)	**0.033**			
Anteroposterior tear size *(mm)*	13.8 ± 6.3 (5.0 to 40.0)	22.2 ± 10.3 (10.0 to 42.0)	**0.004**	14.7 ± 6.5 (5.0 to 38.0)	27.5 ± 8.7 (17.0 to 43.0)	**<0.001**	0.9 ± 4.3 (−20.0 to 14.0)	5.3 ± 6.1 (−1.0 to 15.0)	**0.026**
Mediolateral tear size *(mm)*	14.6 ± 8.7 (5.0 to 46.0)	26.6 ± 8.8 (12.0 to 37.0)	**<0.001**	16.3 ± 9.4 (6.0 to 50.0)	31.6 ± 8.2 (16.0 to 45.0)	**<0.001**	1.7 ± 4.2 (−11.0 to 22.0)	5.0 ± 8.7 (−6.0 to 23.0)	0.126
Tear area *(mm*^*2*^*)*	232.6 ± 227.6 (25.0 to 1,242.0)	639.3 ± 410 (132.0 to 1,302.0)	**<0.001**	272.3 ± 251.9 (40.0 to 1,400.0)	879.4 ± 366.4 (304.0 to 1,376.0)	**<0.001**	39.7 ± 113.4 (−466.0 to 357.0)	240.1 ± 323.5 (−19.0 to 976.0)	**0.005**
Tendon length *(mm)*	25.1 ± 7.2 (7.0 to 38.0)	22.1 ± 7.1 (15.0 to 33.0)	0.171	24.01 ± 6.9 (7.0 to 41.0)	16.5 ± 6.1 (9.0 to 30.0)	**<0.001**	−1 ± 3.3 (−10.0 to 7.0)	−5.6 ± 7.5 (−19.0 to 0.0)	**0.049**

* The values are given as the mean and the standard deviation, with the range in parentheses, or as the number of shoulders, with the percentage in parentheses. FI = fatty infiltration, and SSP = supraspinatus.

‡ Significant values are shown in bold.

§ There were 58 shoulders with data in the no progression group and 9 shoulders in the progression group.

### Prediction of Progression of Muscle FI and Definition of Thresholds

An initial mediolateral tear size of ≥20 mm yielded an FI^SSP^ progression rate of 81.8% (OR, 19.0 [95% CI, 3.7 to 98.8]; p < 0.001). An initial FI^SSP^ of ≥9.9% yielded a progression rate of 72.7% (OR, 17.5 [95% CI, 3.9 to 78.4]; p < 0.001), and an initial anteroposterior tear size of ≥17 mm yielded a progression rate of 72.7% (OR, 8.0 [95% CI, 1.9 to 33.6]; p = 0.003) (Table VI; see also Appendix). Combining these parameters in a multiple logistic regression analysis led to an AUC of 0.913 (95% CI, 0.823 to 1.00). The Hosmer-Lemeshow test confirmed the goodness of fit of the model (p = 0.554). Our model had a positive predictive power of 83.3% and a negative predictive power of 91.8%.

**TABLE VI:** Prediction of Progression of Muscle FI According to Optimal Thresholds*

Thresholds Quantified on Initial MRI	Rate of FI^SSP^ Progression	OR†	AUC‡	P Value§	Sensitivity	Specificity	Youden Index
≥20-mm mediolateral tear size	81.8%	19 (3.7 to 98.8)	0.852 (0.75 to 0.96)	**<0.001**	81.8%	89.9%	0.566
≥9.9% FI^SSP^	72.7%	17.5 (3.9 to 78.4)	0.832 (0.75 to 0.96)	**<0.001**	72.7%	86.8%	0.504
≥17-mm anteroposterior tear size	72.7%	8.0 (1.9 to 33.6)	0.771 (0.62 to 0.93)	**0.003**	72.7%	75%	0.401

* FI = fatty infiltration, and SSP = supraspinatus.

† The values are given as the OR relative to the opposite condition, with the 95% CI in parentheses.

‡ The values are given as the AUC, with the 95% CI in parentheses.

§ Significant values are shown in bold.

The initial muscle volume could not predict the progression of FI^SSP^.

### Interrater Reliability

The individual FI^SSP^ values, the mean FI^SSP^, and the 3D muscle volume measurements all had good to excellent agreement between the 2 raters (see Appendix).

## Discussion

The most important findings of this study were that FI^SSP^ progressed ≥4.5% in 14% of all analyzed tears after a mean follow-up of a little more than 1.5 years, but in some cases as early as 4 to 5 months. These tears had significantly larger initial tear size, greater tendon retraction, less muscle volume, and higher FI^SSP^ than tears in which FI^SSP^ remained largely unchanged. Three risk factors for relevant progression of FI^SSP^ that can be quantified on the initial MRI scan were identified: ≥20-mm mediolateral tear size, ≥9.9% FI^SSP^, and ≥17-mm anteroposterior tear size. These critical thresholds led to a higher risk of relevant progression of FI: 19 times higher risk for ≥20-mm mediolateral tear size, 17.5 times higher risk for ≥9.9% FI^SSP^, and 8 times higher risk for ≥17-mm anteroposterior tear size. Combining them yielded an AUC of 0.913, suggesting a 91% chance of ≥4.5% progression of FI^SSP^ within a mean of 19.5 months.

Understanding the behavior of fatty degeneration of the rotator cuff muscles is crucial, given the known, detrimental correlation between FI and retear rates as well as shoulder function after rotator cuff repair^44-48^ and considering that, once established, FI is irreversible and usually progressive^2,3,24^. More precisely, FI graded as Goutallier stage ≥2 is associated with a definitive loss of muscle function, an increased risk of repair failure, and worse postoperative outcome^4,41,49^. So far, the Goutallier grading system has not been translated into quantified measurements of FF. Using our data and data from the literature^4,31,41,49^, we propose a classification system that defines <8% FF as normal, 8% to <13.5% as advanced, and ≥13.5% as pathological.

We found that approximately 1 in every 4 patients already had advanced or pathological FI^SSP^ at the initial presentation. This can be explained by the fact that both degenerative and traumatic tears were included. It must be assumed that, in the degenerative setting, the onset of the tear occurred sometime before the onset of symptoms. Melis et al.^50^ showed that, in traumatic tears, the mean delay between the occurrence of the tear and grade-2 FI was 3 years. This is somewhat in line with our data, where FI^SSP^ either progressed from normal to advanced in a little less than 2 years or from advanced to pathological in a little less than 1 year.

Studies have described the natural history of conservatively treated rotator cuff tears in terms of tear enlargement and FI, but without quantitative FI assessment or definition of risk factors^7,19,22,51^. Some studies have investigated risk factors for tear enlargement^8,10,18,22,23^, but few have investigated risk factors for the progression of FI^1,10,50^. Identified risk factors for progression of FI are age of >60 years^10,50^, greater initial tear size^1,10,50^, tear enlargement^1,10^, and anterior cable involvement^1,10^; anterior cable involvement has been debated in the literature in which some authors believe that it is a relevant risk factor and other authors do not. It seems to be a surrogate for tear size in that the larger the size of the tear, the more likely that the anterior cable is involved^1,10^. The main limitation of these studies was that FI was evaluated by either ultrasound^1,10^, a CT scan (82%), or a standard 2D MRI scan (18%)^50^ and could therefore be assessed only by qualitative methods.

Our data are in keeping with previous reports, in which the initial tear size and FI seem to play a particularly important role in the prediction of progression of FI. The initial tear size, tendon retraction, FI^SSP^, muscle volume, tear enlargement, and loss of tendon length differed significantly between tears that had relevant progression of FI^SSP^ and tears that did not. However, in the logistic regression analysis, only initial tendon retraction, FI^SSP^, and anteroposterior tear size were risk factors that predicted relevant progression of FI^SSP^. Two other characteristics that have been inconsistently mentioned as risk factors in the literature, age and anterior cable involvement, did not reach significance.

The current study differs from previous investigations as it is the first, to our knowledge, to quantitatively describe the natural history of the FI of the SSP and identify risk factors, with cutoff values, for relevant progression of FI. Quantitative assessment using the arithmetic mean of measurements on 3 MRI slices provides an accurate representation of global FI of the assessed muscle^39^ and good to excellent intrarater and interrater reliability compared with qualitative single-plane assessments^33,39^. Risk factors and cutoff values that can easily be measured on an initial MRI scan equip the clinician with the means to accurately advise the patient on the necessity of surgical treatment or a low-risk time frame for conservative treatment, and can therefore offer valuable help in the decision-making process. Tears that do not meet the established cutoff values can most likely be managed without surgical intervention over 12 months with a high level of assurance.

This study was not without limitations. First and foremost, our work was an analysis of only radiographic factors, without consideration of clinical parameters. Whether a progression of FI^SSP^ that we considered relevant has a clinical influence on, for example, strength or postoperative outcomes cannot be answered. A high number of small and medium tears were included, which may reflect a selection bias: large tears or tears in which the surgeon believed that there was a high risk of progression were more likely to be treated surgically and therefore were not part of this analysis. This could also explain the lower mean SSP FF values in this study compared with other studies^39^. Further limitations were the limited sample size, restricted variation, and prospect for clinical truncation to confound results. Conservative treatment with corticosteroid injections could have influenced FI^SSP^. The time interval between MRI scans was determined by the surgeon, often also depending on the subjectively perceived risk of progression, which could have also influenced the results. There were differences in the follow-up period between the groups with and without relevant FI^SSP^ progression, but these were not significant. We have attempted to base the classification of normal, advanced, and pathological FI^SSP^, and the definition of relevant progression, on the literature and to justify it logically, but, ultimately, this will need to be confirmed or adapted in future work. Due to these limitations, this study should be considered as hypothesis-generating and not definitive.

In conclusion, the results of our study suggest that rotator cuff tears with ≥9.9% FI^SSP^, ≥20-mm mediolateral tear size, and/or ≥17-mm anteroposterior tear size are at high risk for relevant progression of FI within a few months and may be more amenable to early surgical management than rotator cuff tears that do not meet these criteria. However, future studies are needed to define thresholds for preoperative FI leading to rotator cuff repair failure.

## Appendix

Supporting material provided by the authors is posted with the online version of this article as a data supplement at jbjs.org (http://links.lww.com/JBJS/H900).
